# Integrating genetic regulation and schizophrenia-specific splicing quantitative expression with GWAS prioritizes novel risk genes for schizophrenia

**DOI:** 10.1038/s41398-025-03633-8

**Published:** 2025-10-06

**Authors:** Xiaoyan Li, Lingli Fan, Yiran Zhao, Yuanyuan Li, Junyang Wang, Shengmin Xu, Junfeng Xia

**Affiliations:** 1 Information Materials and Intelligent Sensing Laboratory of Anhui Province, Institute of Health Sciences and Technology, Anhui University, Hefei, Anhui China; 2 Key Laboratory of Computational Neuroscience and Brain-Inspired Intelligence (Fudan University), Ministry of Education, Shanghai, China; 3 Department of Human Anatomy, School of Basic Medical Sciences, Zhengzhou University, Zhengzhou, Henan China

**Keywords:** Comparative genomics, Schizophrenia

## Abstract

Alternative splicing (AS) plays a vital role in the pathogenesis of schizophrenia (SCZ). Previous studies have linked the genetic signals from genome-wide association studies (GWAS) with expression quantitative trait loci (eQTL), but the interplay with other genetic regulatory mechanisms, particularly splicing QTL (sQTL), remains unclear. Here, we constructed a comprehensive disease-specific sQTL map to provide genetic variants that could alter gene activity through RNA splicing in SCZ. We analyzed data from 539 SCZ patients, identifying a total of 24,810 significant sQTLs (FDR < 0.05) involving in AS events of 7083 unique genes. By combining this with a large-scale SCZ GWAS, we employed Mendelian randomization (MR) and colocalization analyses to pinpoint 27 significant risk genes with genetic AS regulation that may play a causal role in SCZ. Additional differential splicing analysis of these genes in 539 cases and 754 controls revealed 12 significant genes that may increase SCZ risk due to their AS dysregulation. Notably, five genes (*DPYD*, *LACC1*, *CCDC122*, *ANAPC7*, and *DGKZ*) showed consistent splicing regulation effects in both MR analysis and differential splicing analysis. Pathway enrichment analysis of differentially spliced genes revealed potential biologically pathways relevant to SCZ, particularly in synaptic transmission and microtubule movement. Furthermore, single-cell RNA-seq analysis revealed that several genes were preferentially expressed in specific brain cell types, including oligodendrocytes, microglia, and excitatory neurons. Overall, our findings highlight several susceptibility genes that may contribute to SCZ risk by AS regulation. Further characterization of these genes could advance mechanistic understanding and therapeutic discovery for SCZ.

## Introduction

Schizophrenia (SCZ) is a severe and complex psychiatric disorder characterized by abnormalities in cognition and thought, with a worldwide lifetime prevalence of around 1% [1–3]. Due to the high morbidity and mortality, SCZ imposes enormous economic and medical burdens on individuals, families and societies [4, 5]. However, the pathogenic mechanism of SCZ development is still largely unclear, and existing therapeutic treatments have shown limited benefits [6]. Therefore, there is an urgent need to identify effective and specific biomarkers for the development of therapeutic strategies for SCZ. The heritability of SCZ is estimated as being at least 80%, indicating that genetic factors play a dominant role in the pathogenesis of SCZ [7]. The emergence of the genome-wide association study (GWAS) has created an unprecedented opportunity to dissect the genetic etiology s of SCZ. Over the past decade, GWAS has identified hundreds of risk loci associated with SCZ [8–11]. However, interpretation of the GWAS findings into biology insights and clinical applications remains a great challenge.

Alternative splicing (AS) of pre-mRNA is an essential step in the post-transcriptional gene regulation that removes intronic sequences and links exons specifically [12–14]. Over 95% of multi-exon genes in humans are subjected to AS, greatly increasing transcriptome and protein diversity [12, 15]. Most recent studies have shown that AS is widely present in the nervous and immune systems, and aberrant AS is associated with a variety of brain disorders, especially in SCZ [16–21]. Therefore, unraveling the regulation mechanisms of AS is essential to better understand the pathogenesis of SCZ. Existing evidence indicates that AS regulation can be controlled by genetic variants, and splicing quantitative trait loci (sQTL) has been widely used to explore genetic variants of AS regulation underlying human disease [22–26].

Our current understanding of genetic variants that affect AS and their underlying pathogenic mechanisms in SCZ is still limited. Therefore, integrative approaches that combine sQTL information with GWAS findings have emerged and shown promise in exploring the potential risk genes whose splicing expression levels are affected by the identified risk variants. Mendelian randomization (MR) is a representative integrative approach that uses risk variants associated with splicing quantitative expression as instrumental variables (IVs) to infer the causal influence of an exposure (i.e., risk gene affected by RNA splicing regulation) on an outcome (i.e., disease) [27–29]. By integrating the GWAS genetic findings and sQTL data, MR could infer risk genes that may have a causal role in SCZ. Furthermore, given that RNA splicing is highly heterogeneous in the brain [30], sQTL data based on non-target disease samples may obscure the role of disease-specific AS regulation, resulting in important biological insights that would be missed. Therefore, MR integrative analysis using disease-specific sQTL data will provide new insights into the disease-specific AS regulation mechanism.

To the best of our knowledge, there have been no disease-specific sQTL resources for SCZ. In this study, we first collected 539 SCZ samples with both genotype and transcriptome from two public consortiums and performed a genome-wide sQTL analysis to identify genetic variants that affect AS. In total, we identified 24,810 significant sQTL single-nucleotide polymorphisms (SNPs) using stringent filtering criteria. Furthermore, we performed a comprehensive MR study by integrating the identified SCZ-specific sQTL data with GWAS of SCZ, and proposed 27 significant genes whose genetically AS regulation may have a causal role in SCZ. By combining evidence of colocalization and differential splicing analysis, we identified 12 promising risk genes for SCZ. In addition, the single-cell transcriptomic analysis revealed that 13 genes are enriched in brain cell types, including oligodendrocytes, inhibitory neurons, excitatory neurons, astrocytes, and microglial cells. Collectively, our study offers a comprehensive resource of SCZ-specific sQTL map and provides a set of promising novel drug targets with strong evidence for SCZ.

## Materials and methods

### SNP genotyping and RNA sequencing (RNA-seq) data of SCZ participants

We used the SNP genotyping and RNA-seq data in brain tissue from two cohorts, including the CommonMind Consortium (CMC [31]) and the Lieber Institute for Brain Development (LIBD [32, 33]). Briefly, all quality-controlled DNA genotyping and raw RNA-seq files for the dorsolateral prefrontal cortex (DLPFC) region of the human brain were downloaded from the LIBD database (http://eqtl.brainseq.org) and the CMC portal (https://www.synapse.org/CMC). Eventually, a total of 539 SCZ cases from the CMC (N = 328) and LIBD (N = 211) datasets were included in this study, consisting mainly of European participants (Supplementary Table 1). For genotyping data, the downloaded DNA genotyping files from each SCZ individual was subsequently merged with PLINK v1.9 [34] and totaling 27,332,850 genotyped or imputed markers were used for the following sQTL analysis. For RNA-seq data of LIBD, the downloaded RNA-seq FASTQ data were cleaned using fastp v0.23.4 [35] and then aligned to the GRCh37 genome assembly by STAR v2.5.2a [36] (converted into BAM files). Furthermore, the obtained BAM files of LIBD were merged with the CMC downloaded BAM files, and the WASP [37] tool was then employed to remove reads with potential mapping bias. More detailed information about the sample collection, DNA and RNA extraction and sequencing, quality control and statistical analyses have been described in previous publications [31–33].

### Splicing quantification

To determine splicing quantification on RNA-seq data from DLPFC brain regions of 539 SCZ participants, we chose to quantify AS events using the unannotated LeafCutter [38] algorithm. The intron usage rates (i.e., percentage spliced-in value (PSI) value) calculated by LeafCutter were used as the splicing quantification indicator in this study. Specifically, we converted the BAM files into an intron junction file by using the bam2junc.sh script from LeafCutter. Using the leafcutter_cluster.py script, intron clustering was then performed with the following parameters: “-minclureads 50, -mincluratio 0.001, and -maxintronlen 500000”. We mapped intron clusters to genes based on exon coordinates from GENCODE v.19 annotation and the introns present in more than 40% of all samples were selected for further analysis. The PSI values were computed using the prepare_phenotype_table.py script from LeafCutter for the qualifying introns.

### Identification of SCZ sQTLs

To identify sQTL SNPs in SCZ, we conducted a cis-sQTL analysis (within 1000 kb up/downstream of the intron clusters) of the obtained PSI matrix and genotype data. With the linear regression models implemented in the FastQTL [39] software package, the association between PSI values of AS events and SNP genotypes (i.e., sQTLs) was examined. To control for potential confounders (such as genetic, biological, and technical factors), we performed covariance correction analysis to regress out relevant covariates, including age, sex, RNA integrity number, population structure, and sequencing library batch effects. More details on the sQTL analyses were given in our previous work [17]. The intron level sQTL *P*-values were obtained by applying the permutation procedure (adaptively permute 1000 times) from FastQTL, Finally, sQTLs with Benjamini-Hochberg correction (FDR < 0.05) were considered statistically significant.

### SCZ GWAS

To maximize statistical power, summary genetic association data from the largest available GWAS of SCZ [40] were used as outcome data in further MR analysis. Summary level data for 7,659,767 SNPs were obtained from the Psychiatric Genomics Consortium (PGC) data portal. Briefly, Trubetskoy et al. performed a large-scale trans-ancestry SCZ GWAS consisting of 74,776 SCZ cases and 101023 control individuals, which included European, Asian, African American, and Latino ancestry populations. Ultimately, they reported a total of 342 genome-wide independent significant SNPs located in 287 distinct genomic regions. To avoid biases due to variations in LD and allele frequencies, only GWAS from European populations (53,386 cases and 77,258 controls) were considered in this study. Detailed information on sample collection, genotyping, quality control, and statistical analyses can be found in the original publication [40] and the PGC website (https://www.med.unc.edu/pgc).

### MR analysis

Genetic variation associated with RNA splicing was used as an IV to assess the causal association between exposure (i.e., SCZ sQTL data) and outcome (i.e., SCZ GWAS data). MR analysis was conducted using the “TwoSampleMR” R package (version 0.5.6) [41]. Prior to the MR analysis, we harmonized the exposure and outcome data to ensure the same effect allele of the SNP was used in both the sQTL and GWAS datasets. IVs were then performed linkage disequilibrium (LD) clumping using a window of 5000 kb and a low LD (r^2^ < 0.01) between IVs to ensure that the IVs (i.e., SNPs) were independent. MR analyses employ the Wald ratio method when only one cis IV is considered, and the inverse variance weighted (IVW) method when two or more cis IV are considered. Specifically, IVW combines the Wald ratio estimates of each individual SNP into one causal estimate for each risk factor. As our work included only one IV, we did not undertake any sensitivity analyses [42, 43]. To account for multiple testing, a Bonferroni correction was applied to adjust for 5179 independent tests (0.01/5179, *P* = 1.93 × 10^−6^, 5179 is the number of effective splicing sites valid AS events used for MR analyses). More details of the MR analysis can be found in the original papers [44, 45].

For the MR analysis, the same IV (i.e., SNP) should influence both exposure factor and outcome factor, rather than sharing IVs coincidentally due to LD. To assess the probability of the same IV being responsible between SCZ and sQTL, we further performed colocalization analysis for SCZ risk using the Bayesian approach implemented in the R package Coloc v.5.1.0 [46] (https://github.com/chr1swallace/coloc). Specifically, colocalization analysis was conducted to adjust such spurious results and posterior probabilities for five hypotheses (H0, H1, H2, H3, H4) were calculated. The correct hypothesis above is H4, and PPH4 (posterior probability for hypothesis 4) specifically quantifies the probability that both traits are driven by the same causal variant within a splicing region. To ensure the reliability of the MR results, we set a strict significant threshold for the posterior probability (two significant associations sharing a common causal variant) at PPH4 > 0.90 in colocalization analysis. Further details about the principle of the colocalization analysis have been published previously [45].

### Differential splicing analysis

To investigate the RNA splicing level of the MR significant results in SCZ cases compared with controls, we obtained publicly available transcriptome RNA-seq data of 539 SCZ cases and 754 controls from CMC and LIBD datasets. The LeafCutter [38] was employed to generate PSI matrices with the same processing procedure as previously described [17]. Then, differential splicing analyses was carried out using the Wilcoxon rank sum test to compare PSI values (i.e., the RNA splicing level) between SCZ cases and controls, and the *P*-value < 1 × 10^−3^ was considered significant. More information on sample and RNA-seq handling protocols can be found in the original publication [31–33].

### Functional enrichment analysis

There was no significant correlation the MR-identified genes after applying the Bonferroni correction, which is likely due to the assumption of independent tests that this correction requires. Thus, the top 500 MR results with the smallest *P*-value were selected for the functional enrichment analysis, allowing for more genes to be included. We used the clusterProfiler package in R language (version 4.10.0) for functional enrichment analysis, including Gene Ontology (GO) and Wiki Pathways (WP) gene sets pathway enrichment analysis. For the enrichment calculation of Biological Processes, Human (org.Hs.eg.db) gene annotation with Entrez Gene identifiers was used.

### single-cell RNA-seq (scRNA-seq) analysis

To explore if MR-significant genes were specifically expressed in specific brain cell populations, we performed a single-cell expression analysis. First, we downloaded raw scRNA-seq data (i.e., FASTQ files) of 24 cognitively normal individuals in the PFC brain region from Mathys et al. study [47] (https://www.synapse.org/#!Synapse:syn18485175). Furthermore, Seurat [48] (version 5.0.3) workflow was applied to scRNA-seq data for data preprocessing and analysis, including gene and cell quality control, normalization and transformation, and cluster annotation. Specifically, genes expressed in fewer than 3 cells and cells expressing less than 200 genes were excluded. To reduce noise and improve interpretability, we performed principal component analysis (PCA) for all highly variable genes. We employed the FindAllMarkers function in Seurat to find marker genes for each cluster. Our study focused on 6 brain cell types provided by Mathys et al. [47], including oligodendrocytes, inhibitory neurons, excitatory neurons, astrocytes, microglial, and oligodendrocyte progenitor cells. To determine if potential SCZ-causal are highly expressed in one particular cell type, we explored the cell-type specific expression of these genes using the Wilcoxon rank sum test. To control the false discovery rate, FDR correction was applied to all the genes analyzed, and genes with FDR < 0.05 were considered significant.

### MR analysis in non-SCZ study participants

We further examined whether our MR findings are informative for non-SCZ study populations using sQTL data of non-SCZ populations from the Genotype-Tissue Expression (GTEx) project. Briefly, the GTEx project characterized and released sQTLs in 54 tissues of over 900 healthy individuals. We have downloaded the latest sQTL data (Brain_Frontal_Cortex_BA9.v10) of PFC brain tissues (N = 268) from GTEx v10 and performed MR analysis using identical pipelines and parameters as in our SCZ MR study. The threshold for significant associations with MR evidence was set at P < 2.66 × 10^–6^ (i.e., Bonferroni corrected *P*-value cutoff of 0.01/3764 effective splicing sites).

## Results

### Identification and characterization of SCZ-specific sQTLs

**Fig. 1:**
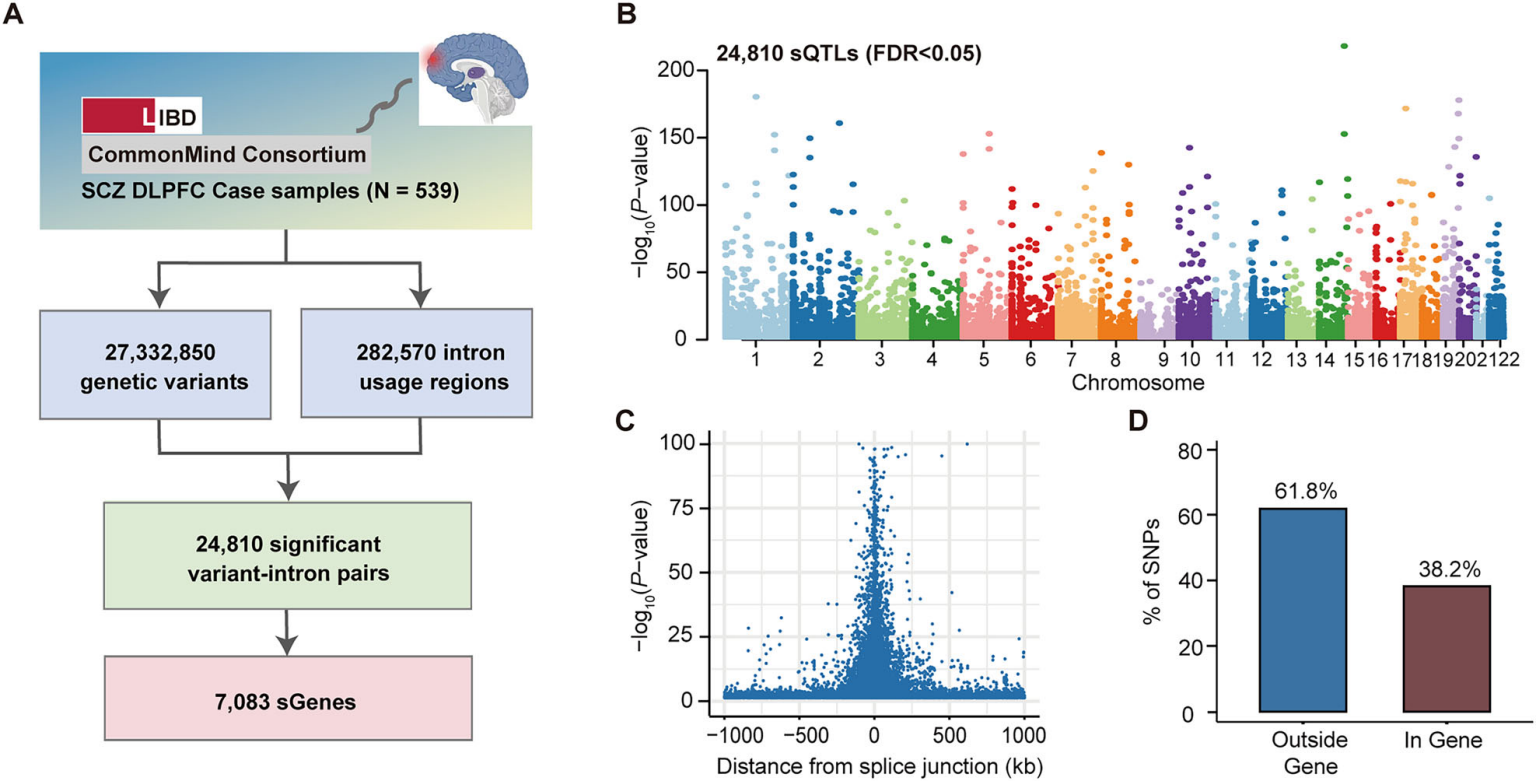
Identification and characterization of sQTLs. **A** The pipeline of sQTL discovery is based on 539 SCZ brain tissues from CMC and LIBD datasets. **B** The Manhattan plot shows the distribution of these sQTL SNPs on different chromosomes. **C** Position of sQTL SNPs in relation to the splice junction. **D** Percentage (%) of index sQTL SNPs (the most significant variant within per intron usage region) located in or outside the corresponding sGene.

To investigate the disease-specific genetic control of RNA splicing in SCZ, we performed a genome-wide cis-sQTL analysis using 539 SCZ samples with both PSI matrix of AS events and genotype from CMC and BrainSeq datasets (Fig. 1A). We harvested a total of 282,570 AS events and 27,332,850 genotyped SNPs were retained for further sQTL analysis after stringent quality control. Eventually, we identified 24,810 significant sQTL SNPs (FDR < 0.05) involving 7083 unique sQTL-harboring genes (sGenes) in brain PFC regions of SCZ samples (Fig. 1B and Supplementary Table 2**)**. To investigate the genomic distribution of sQTL SNPs, we examined the distance between a sQTL SNP and the corresponding nearest splicing junction. Consistent with previous findings [49–51], sQTL SNPs were enriched around the splice junction (Fig. 1C**)**. In addition, we observed that roughly 38.2% of sQTL SNP were located within the body of the gene where the corresponding AS event occurred (Fig. 1D**)**.

### MR analysis using SCZ-specific sQTL data identified 27 candidate SCZ susceptibility genes

**Fig. 2:**
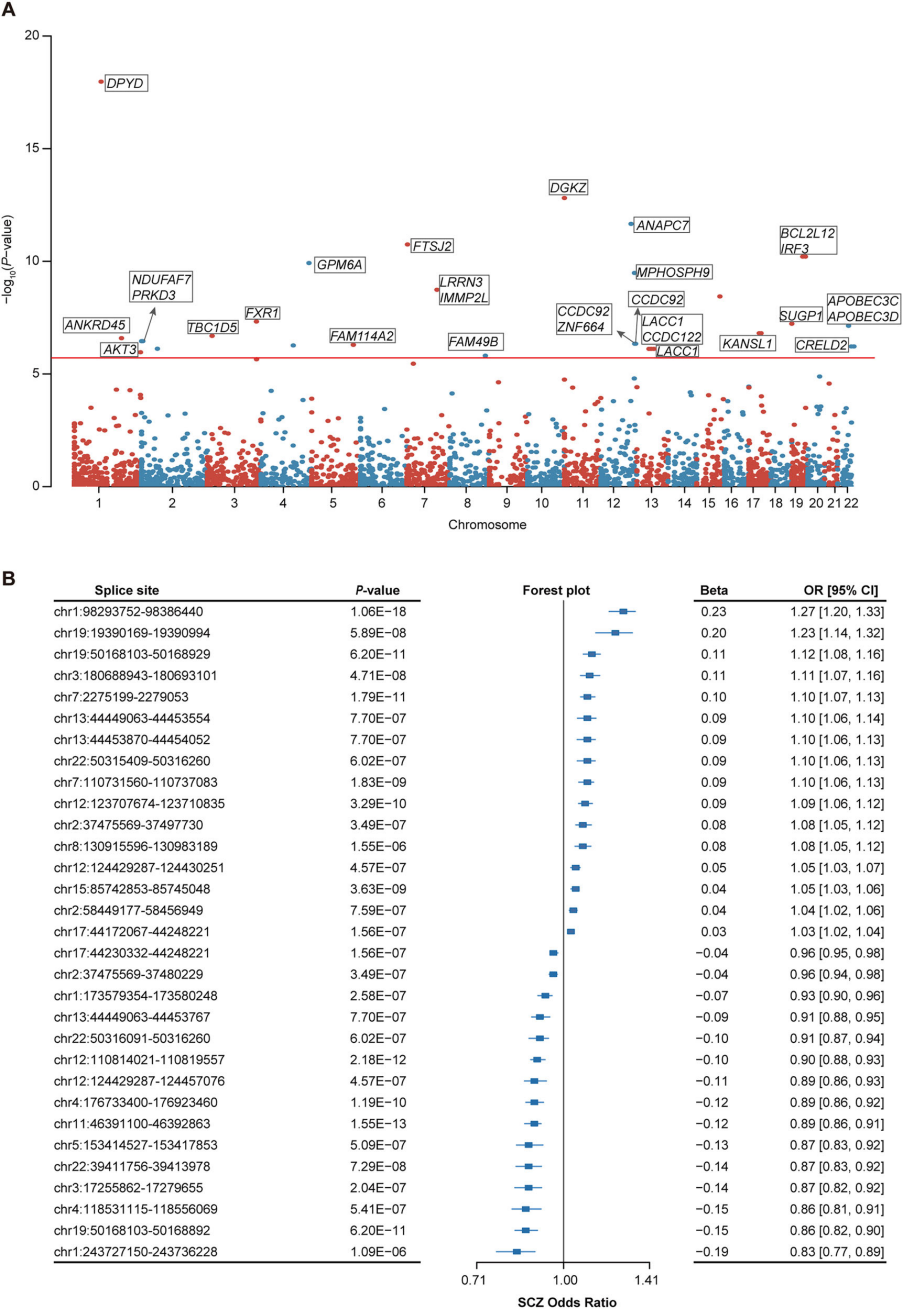
MR Analysis using SCZ-specific sQTL data identified 27 significant risk genes for SCZ. **A** The Manhattan plot shows all significant sGenes within intron usage regions in MR analysis. The red line indicates the *P* threshold for Bonferroni correction. **B** The forest plot shows the results that 31 intron usage regions reached significance in MR analysis. Additionally, we calculated 95% confidence interval for the odds ratio. 95% CI 95%: confidence interval, OR odds ratio, SCZ schizophrenia.

To identify susceptibility genes that causally contribute to SCZ risk by affecting RNA splicing, we performed a SCZ-specific MR study through integrating SCZ GWAS summary genetic data (53,386 cases and 77,258 controls) with SCZ-specific sQTL data (N = 539 SCZ samples). Notably, Wald ratio estimates were exclusively applied in our final MR analysis because only one qualified SNP per splicing region survived rigorous IV selection criteria (minor allele frequency > 0.01, LD pruning r² < 0.01, and F-statistic > 10). This scenario inherently necessitates the Wald ratio approach (the statistically optimal method when a single IV is available), as it provides unbiased causal effect estimates without requiring instrument homogeneity assumptions essential for IVW meta-analysis. Therefore, the MR associations with *P*-value < 1.93 × 10^−6^ were considered statistically significant after Bonferroni correction for multiple tests. We found that all MR significant results were robust to colocalization analyses (PPH4 > 0.90). Consequently, we identified 27 genes within 31 intron usage regions that demonstrated a significant association with SCZ risk, supported by robust evidence (Fig. 2A and Supplementary Table 3). Among which, one significant gene (*DPYD*) at chr1:98293752–98386440 intron usage region showed the most significant association (*P* = 1.06 × 10^−18^). Other significant potential susceptibility genes include *DGKZ*, *ANAPC7*, *FTSJ2*, *BCL2L12*, *IRF3*, *GPM6A*, *MPHOSPH9*, *LRRN3*, and *IMMP2L*. Interestingly, genetically raised splicing quantitative expression (i.e., PSI values) of gene *KANSL1* was associated with reduced SCZ risk in chr17:44230332–44248221 splicing site (OR = 0.97; *P* = 1.56 × 10^–7^) and increased SCZ risk in chr17:44172067–44248221 splicing site (OR = 1.03; *P* = 1.56 × 10^–7^) (Fig. 2B and Supplementary Table 3), indicating that distinct intron usage regions of the same gene have different biological functions in SCZ.

In addition, by comparing MR significant results with the largest SCZ GWAS from PGC3, we found that 19/27 significant genes identified by MR were located at known SCZ susceptibility loci, including *DPYD*, *DGKZ*, *BCL2L12*, *IRF3*, *MPHOSPH9*, *LRRN3*, *IMMP2L*, *FXR1*, *SUGP1*, *KANSL1*, *ANKRD45*, *TBC1D5*, *NDUFAF7*, *PRKD3*, *FAM114A2*, *LACC1*, *CCDC122*, *AKT3*, and *GPM6A*. These overlapping MR results affected by AS might help to pinpoint potential target genes in each GWAS signal. More importantly, we found that 8/27 genes with AS events did not overlap with known association loci of SCZ, including *FTSJ2*, *CSPG4P12*, *CCDC92*, *ZNF664*, *CRELD2*, *FAM49B*, *APOBEC3C*, and *APOBEC3D*. These results indicated that incorporating SCZ-specific sQTL data might facilitate the identification of novel target genes beyond GWAS findings.

### Splicing dysregulation of 16 genes within 10 intron usage regions identified by MR analysis in SCZ cases

**Fig. 3:**
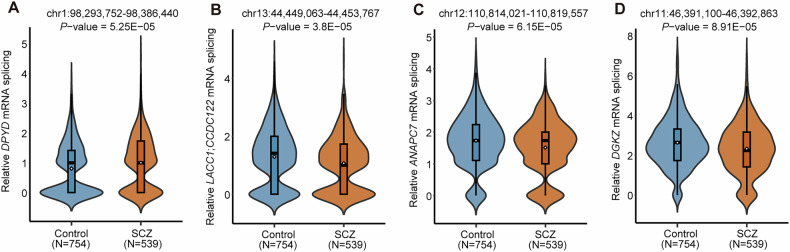
Differential splicing analysis of the four MR-identified significant intron usage regions. Violin plots show splicing dysregulation of four intron usage regions (**A-D**) in SCZ cases compared with controls. SCZ schizophrenia.

Significant intron usage regions predicted in MR analyses whose genetically AS regulation might have essential roles in SCZ. After excluding, a total of 539 SCZ cases and 754 healthy controls from CMC and BrainSeq datasets were included in the differential splicing analysis for SCZ. Among the 31 significant intron usage regions, we observed that 10/31 intron usage regions (corresponding to 12 sGenes) were differentially splicing quantitative expression (nominal *P*-value < 1 × 10^−3^) in SCZ cases compared with controls (Supplementary Table 4), suggesting that these overlapping intron usage regions represent promising functional genetic loci for SCZ. Notably, MR Analysis revealed that the upregulation of splicing expression in one intron usage region is associated with an increased risk of SCZ (OR > 1.00) and the upregulation of splicing expression in three intron usage regions is associated with a decreased risk of SCZ (OR < 1.00). These regions included chr1:98293752–98386440 (corresponding to sGene *DPYD*), chr13:44449063–44453767 (corresponding to sGene *LACC1* and *CCDC122*), chr12:110814021–110819557 (corresponding to sGene *ANAPC7*) and chr11:46391100–46392863 (corresponding to sGene *DGKZ*). Coherently with these predictions, differential splicing analysis validated the changes in splicing expression for the above four splicing regions in SCZ cases. Collectively, these expression data provided consistent and convergent evidence in favor of five genes affected by RNA splicing that may have a major role in SCZ, including *DPYD*, *LACC1*, *CCDC122*, *ANAPC7* and *DGKZ* (Fig. 3A-D). In addition, we found that the remaining seven genes had different directions of effect between MR and differential splicing analysis, possibly due to the biological heterogeneity of different tissues used in sQTL and GWAS datasets.

### Functional enrichment analysis of the identified MR genes revealed related biological processes

**Fig. 4:**
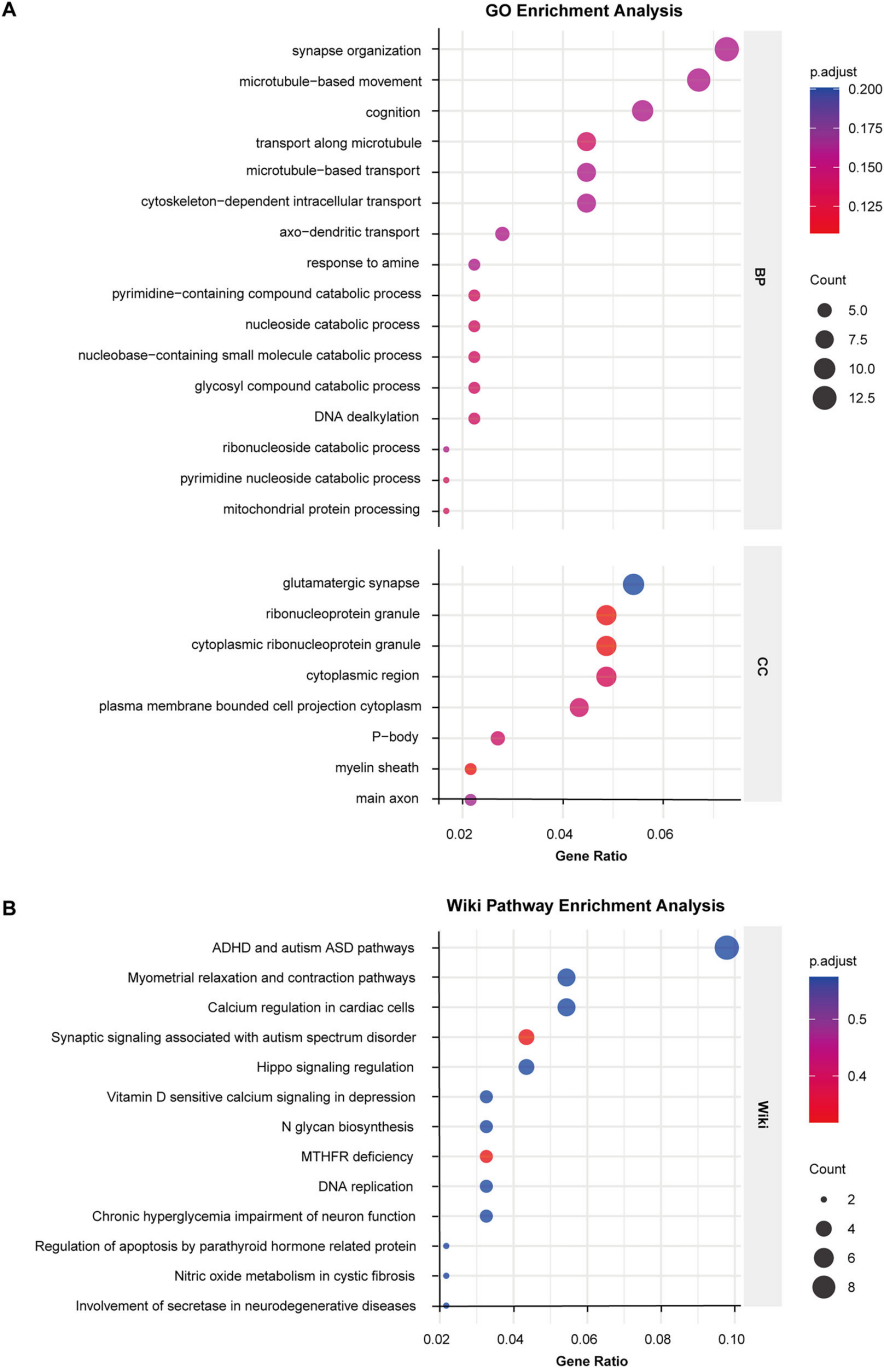
GO and Wiki pathway enrichment analysis of MR-identified risk genes. **A** GO pathway enrichment analysis of MR-identified risk genes. **B** Wiki pathway enrichment analysis of MR-identified risk genes.

To get insights into the biological processes regulated by the top 500 MR-derived genes (ranked according to *P*-value), we conducted functional enrichment analysis using two different programs: GO term and Wiki pathway enrichment analysis. Specifically, GO enrichment analysis revealed a strong enrichment of MR genes in biological processes like synapse organization, microtubule−based movement, cognition, and transport along microtubule (Fig. 4A). Among these, the most significantly enriched pathways of the GO analysis were synaptic organization. This finding aligns with previous reports of strong genetic associations between synaptic function and the pathology of SCZ [52–54], providing independent support for synaptic development as a key process disrupted in SCZ risk. Wiki enrichment analysis further revealed that the ADHD (attention deficit hyperactivity disorder) and ASD (autism spectrum disorder) pathways and synaptic signaling associated with ASD (Fig. 4B). This result is not unexpected, given the increasing body of evidence indicating a shared genetic risk among ADHD, ASD, and SCZ [55–58].

### Cell-type specific expression of the potential SCZ-susceptibility genes

**Fig. 5:**
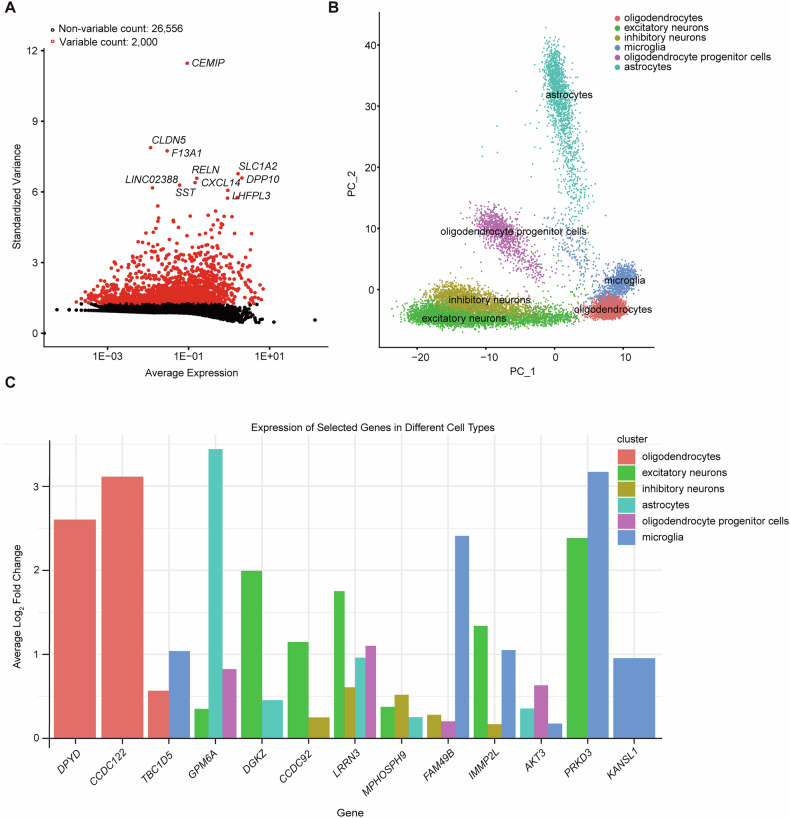
Single-cell sequencing analysis of the 27 MR-identified risk genes in the human brain. **A** Dot plot of top 2000 hypervariable genes for brain cell type. The top ten genes were flagged. **B** UMAP plot shows six cell clusters. **C** Enrichment of the MR-identified risk genes in different cell types of the brain.

To investigate the expression of SCZ-susceptibility genes in various brain-relevant cell types, we analyzed the activity of these genes across different cell types using scRNA-seq data from the PFC brain region of cognitively normal individuals. Clustering analysis on scRNA-seq data was performed using the Seurat pipeline to identify cell types or subpopulations. We selected 2000 hypervariable genes and the top ten genes were flagged (Fig. 5A). Furthermore, we identified six clusters of different brain cell types (Fig. 5B). Among 27 SCZ-susceptibility genes identified in MR analysis, 13/27 (*DPYD*, *CCDC122*, *TBC1D5*, *GPM6A*, *DGKZ*, *CCDC92*, *LRRN3*, *MPHOSPH9*, *FAM49B*, *IMMP2L*, *AKT3*, *PRKD3* and *KANSL1*) were enriched in one or more cell types, including oligodendrocytes, inhibitory neuron, excitatory neurons, astrocyte, and microglial (Fig. 5C). Of note, three genes show evidence of enrichment in a particular cell type, including *DPYD*, *CCDC122*, and *KANSL1*. For instance, the genes *DPYD* and *CCDC122* were highly expressed in oligodendrocytes, as shown in Fig. 5C.

## Discussion

Hitherto, GWAS has identified more than 200 risk loci for SCZ, but it is unclear how they confer SCZ risk. More importantly, RNA splicing has been reported to play a key role in the development of SCZ. Considering that sQTL has not been well characterized in SCZ cases, we systematically undertook a genome-wide sQTL analysis using genotype and RNA-seq data derived from 539 SCZ samples. To identify the potential risk genes at SCZ risk loci, we further performed a MR integrative study using the obtained SCZ-specific sQTL data with the latest SCZ GWAS results. We identified 27 potential causal SCZ genes within 31 intron usage regions that act via AS regulation to contribute to SCZ pathogenesis (Supplementary Table 5). Moreover, we found that 12 genes have displayed aberrant splicing expression in SCZ cases compared with controls. Of note, five of these genes (*DPYD*, *LACC1*, *CCDC122*, *ANAPC7*, and *DGKZ*; Supplementary Table 6) showed the same directions of effect in both the MR and differential splicing analysis. This strongly indicates that these genes could be potential new treatment targets for SCZ.

One interesting finding in this study was regarding gene *DPYD* associated with the risk of SCZ. Among the 27 significant MR results, *DPYD* (located in chr1:98293752–98386440 intron usage region) showed a strong significant association (*P-*value = 1.06 × 10^−18^), whose genetically AS regulation in PFC brain tissues may have a causal role in SCZ. MR results have shown that genetically increased gene *DPYD* splicing expression was associated with increased SCZ risk in brain tissue (OR = 1.26). By comparing with differential splicing analysis findings, we found that the splicing expression level of *DPYD* was most significantly upregulated in SCZ cases versus normal controls (*P-*value = 5.25 × 10^−5^). These consistent results strongly suggested that *DPYD* represents a potential causal gene for brain SCZ. Moreover, we found that *DPYD* was located within a reported GWAS hit signal, indicating its implication in the latest SCZ GWAS. Furthermore, our scRNA-seq analysis showed that *DPYD* points to specific cell types that they likely act through to oligodendrocytes to SCZ. These lines of evidence support that *DPYD* may be a promising treatment target for SCZ.

It is well established that gene regulation is highly context-dependent, often exhibiting cell-type and developmental stage specificity. Consequently, certain genes may only contribute to schizophrenia genetic risk within specific cellular contexts. For the single-cell enrichment analysis, we found that 13 SCZ susceptibility genes identified by MR analysis were enriched in one or more cell types. For instance, genes *DPYD* and *CCDC122* exhibit specific high expression in oligodendrocytes, indicating that the cell-type-specific expression patterns of these susceptibility genes are closely related to the pathophysiology of SCZ. Increasing evidence indicates abnormal expression of oligodendrocyte-related genes, which may severely impair myelin formation or maintenance [59, 60]. Myelin abnormalities can disrupt the precision and synchrony of synaptic transmission, leading to synaptic dysfunction. Furthermore, such impairment affects normal neural circuit function, ultimately resulting in cognitive, emotional, and behavioral symptoms in SCZ patients [61–63].

To evaluate the extent of sQTL sharing between SCZ and GTEx DLPFC tissues, we first counted the number of sharing sGenes that were significant in both SCZ and GTEx. We observed that 850 (approximately 12%) of the 7083 sGenes in SCZ overlapped those in GTEx PFC brain tissues. In addition, the 6233 non-overlapping sGenes represent potential SCZ-specific regulators (Supplementary fig. 1). Crucially, it is possible that case-specific sQTLs are a result of reverse causation. Considering that differences in LD patterns between populations tend to affect the MR results, we then performed MR analysis using sQTL data of GTEx and SCZ GWAS data and full significant MR results are shown in Supplementary Table 7. Using cis-sQTLs from the GTEx dataset as proposed instruments, we identified 20 genes that reached MR significance. Furthermore, we found that 10/27 replicated genes showed significance in both sQTL datasets (Supplementary Table 7), while the remaining 17 potential risk genes were uniquely significant in our SCZ-specific MR analysis. In addition, 4 of the 10 replicated genes had different directions of the MR effect between tissues. For example, genetically raised gene *DGKZ* expression was associated with reduced SCZ risk in SCZ brain tissues (OR = 0.88; 95% CI, 0.86–0.91; *P-*value = 1.55 × 10^−13^) and increased SCZ risk in GTEx healthy brain tissues (OR = 1.16; 95% CI, 1.12–1.21; *P-*value = 1.32 × 10^–13^). These results suggest that integrating SCZ-specific sQTL data may provide novel insights into the mechanism of SCZ.

It is important to acknowledge the potential limitations of our study. Firstly, the sQTL data and GWAS summary statistics were mainly from individuals of European ancestry, limiting the generalizability of our findings to other populations. Therefore, expanding research studies to evaluate the association of susceptibility genes with SCZ in other ethnic populations seems essential. Secondly, the sQTL dataset used in our MR integration study was primarily from brain tissue of the human brain. External single-cell sQTL datasets should be utilized to investigate the causal genes associated with SCZ. Thirdly, we only included cis-sQTL SNPs as IVs in the MR study to maintain the assumption that IVs must be strongly associated with the exposure. However, this may ignore the complex role of trans-sQTL in the genetic regulation mediated for SCZ. Lastly, the identified SCZ risk genes require further experiment validation to verify their biological function.

In summary, by integrating unique SCZ-specific sQTL data with the latest SCZ GWAS data, we performed MR analysis and identified 27 candidate susceptibility genes that contribute to SCZ risk through AS regulation. Differential splicing analyses further validated these findings, highlighting five potentially causal genes with the same direction of effect that may pose a risk of developing SCZ. We also identified key pathways and brain cell type specificity important in the pathogenesis of the SCZ. Our findings not only advanced our understanding of the pathological mechanisms of SCZ but also provided valuable targets and directions for developing effective treatments.

## Supplementary information

Supplemental information

Supplementary Tables

## Data Availability

All data generated in this study will be available from the corresponding author on reasonable request.
