# Supplementary material for: Integrating genetic regulation and schizophrenia-specific splicing quantitative expression with GWAS prioritizes novel risk genes for schizophrenia
Source: Transl Psychiatry. 2025 Oct 6;15:379. doi: 10.1038/s41398-025-03633-8 (PMC12501019; doi:10.1038/s41398-025-03633-8)
Supplement: Supplementary file 1 — Supplemental information [file 41398_2025_3633_MOESM1_ESM.docx]

**Supplemental information**


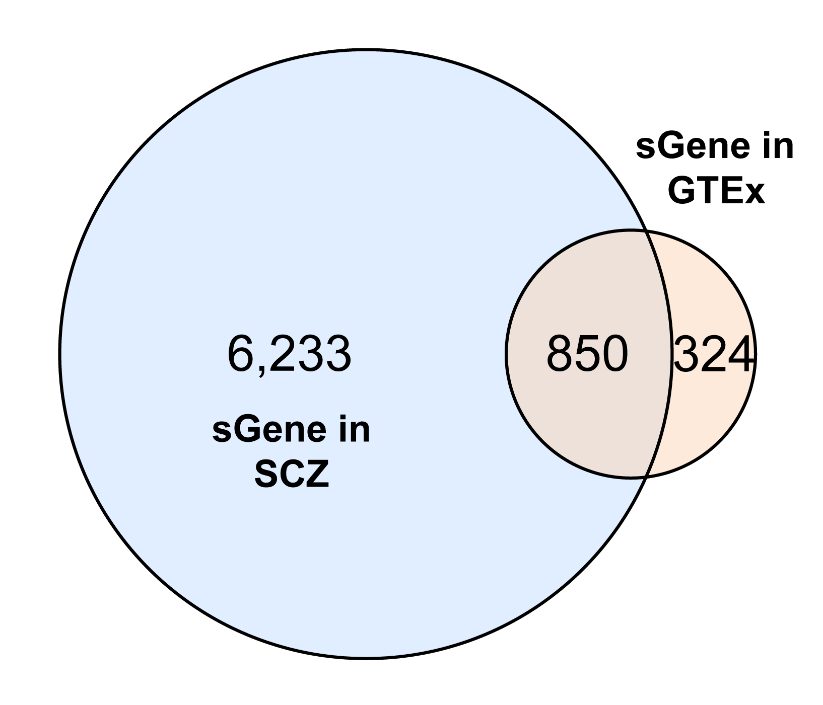


Figure S1. Venn diagram showing the overlap of sGenes between the SCZ and the Genotype-Tissue Expression (GTEx) project.
